# Molecular Recognition of Metal Complexes by DNA: A Comparative Study of the Interactions of the Parent Complexes [PtCl(TERPY)]Cl and [AuCl(TERPY)]Cl	_2_ with Double Stranded DNA

**DOI:** 10.1155/BCA.2005.239

**Published:** 2005

**Authors:** Luigi Messori, Giordana Marcon, Alessio Innocenti, Enzo Gallori, Marco Franchi, Pierluigi Orioli

**Affiliations:** 1 Department of Chemistry University of Florence via della Lastruccia 3 Sesto Fiorentino Florence 50019 Italy; 2 Department of Animal Biology and Genetics “Leo Pardi” University of Florence via Romana 17-19 Florence 50122 Italy

**Keywords:** Gold(III) complexes, platinum(II) complexes, *calf thymus* DNA (*ct*-DNA), pHV14 plasmid, circular dichroism, absorption spectroscopy, DNA melting, ultrafiltration

## Abstract

The interactions of the parent complexes [AuCl(Terpy)]Cl_2_ and [PtCl(Terpy)]Cl with DNA were analysed by various physicochemical methods. Surprisingly, these metal complexes produce different interaction patterns with DNA in spite of their profound structural similarity. Indeed, important modifications are detected in the characteristic UV-Vis bands of [PtCl(Terpy)]Cl upon addition of *ct*-DNA, while the spectrum of [AuCl(Terpy)]Cl_2_ is almost unaffected. Gel electrophoresis studies confirm these findings: [PtCl(Terpy)]Cl — but not [AuCl(Terpy)]Cl_2_ — retards significantly the mobility of the supercoiled form of the pHV14 plasmid after a short incubation time. Ultrafiltration studies indicate that the affinity of [PtCl(Terpy)]Cl for *ct*-DNA is significantly greater than that of [AuCl(Terpy)]Cl_2_. On the other hand, both [AuCl(Terpy)]Cl_2_ and [PtCl(Terpy)]Cl induce important changes in the CD spectrum of *ct*-DNA, at high concentration, and increase its T_m_ value. Remarkably, the analysed metal-complex/DNA interaction patterns depend critically on the incubation times. We propose that [PtCl(Terpy)]Cl quickly intercalates DNA; then, formation of coordinative bonds progressively takes place with time. At variance, [AuCl(Terpy)]Cl_2_ first interacts electrostatically with the DNA surface, with subsequent slow formation of some coordinative bonds.


### Molecular Recognition of Metal Complexes by DNA: A Comparative Study of the Interactions of the Parent Complexes [PtCl(TERPY)]Cl and [AuCl(TERPY)]$Cl_2$ with Double Stranded DNA.

*Luigi Messori[#], *Giordana Marcon, *Alessio Innocenti, [α]Enzo Gallori, [α]Marco Franchi and *Pierluigi Orioli.

[*]*Department of Chemistry, University of Florence, via della Lastruccia 3, 50019 Sesto Fiorentino (Florence), Italy;*

[α]*Department of Animal Biology and Genetics "Leo Pardi", University of Florence, via Romana 17-19, 50122 Florence, Italy.*

### ABSTRACT

The interactions of the parent complexes [AuCl(Terpy)]$Cl_2$ and [PtCl(Terpy)]Cl with DNA were analysed by various physicochemical methods. Surprisingly, these metal complexes produce different interaction patterns with DNA in spite of their profound structural similarity. Indeed, important modifications are detected in the characteristic UV-Vis bands of [PtCl(Terpy)]Cl upon addition of *ct*-DNA, while the spectrum of [AuCl(Terpy)]$Cl_2$ is almost unaffected. Gel electrophoresis studies confirm these findings: [PtCl(Terpy)]Cl – but not [AuCl(Terpy)]$Cl_2$ – retards significantly the mobility of the supercoiled form of the pHV14 plasmid after a short incubation time. Ultrafiltration studies indicate that the affinity of [PtCl(Terpy)]Cl for *ct*-DNA is significantly greater than that of [AuCl(Terpy)]$Cl_2$. On the other hand, both [AuCl(Terpy)]$Cl_2$ and [PtCl(Terpy)]Cl induce important changes in the CD spectrum of *ct*-DNA, at high concentration, and increase its $T_m$ value. Remarkably, the analysed metal-complex/DNA interaction patterns depend critically on the incubation times. We propose that [PtCl(Terpy)]Cl quickly intercalates DNA; then, formation of coordinative bonds progressively takes place with time. At variance, [AuCl(Terpy)]$Cl_2$ first interacts electrostatically with the DNA surface, with subsequent slow formation of some coordinative bonds.

**Keywords:** Gold(III) complexes, platinum(II) complexes, *calf thymus* DNA (*ct*-DNA), pHV14 plasmid, circular dichroism, absorption spectroscopy, DNA melting, ultrafiltration

---

[#] Corresponding author: Dr. Luigi Messori,
via della Lastruccia 3, 50019 Sesto Fiorentino (Florence), Italy
e-mail: luigi.messori@unifi.it, Telephone Number: +39-055-4573284, Fax Number: +39-055-4573385

### INTRODUCTION

There is today great interest in understanding how DNA recognizes metal complexes, particular attention being devoted to non-covalent binding modes /1/. As previously suggested, metal complexes interact non-covalently with double helix DNA in three basic ways: external binding, groove binding and intercalation /2/. External binding refers to those interactions that occur on the DNA surface, mainly governed by electrostatic effects; groove binding is a stronger type of interaction that takes place when a molecule of proper size enters one of the grooves of DNA. Intercalation consists of the insertion of a flat aromatic molecule between two adjacent bases.

The interactions of various platinum terpyridine complexes with DNA have been extensively investigated in the last three decades, mainly by the groups of Lippard /3-7/, McMillin /8-10/, Romeo /11-13/ and Lowe /14,15/. Interest in these compounds is related, at least in part, to their promising pharmacological properties, specifically as potential antitumor and anti-tripanosomial agents. On the ground of the large body of experimental evidence collected so far it has been argued that square planar platinum terpyridine compounds bind DNA predominantly by intercalation /3-7/. The resulting duplex unwinding angle is comparable to that measured following DNA intercalation by ethidium bromide /16/. The [PtCl(Terpy)]Cl shows a binding constant for *ct*-DNA of about $1x10^5$; remarkably this complex first intercalates DNA, then slowly forms covalent links to the nucleobases /10, 17/.

[AuCl(Terpy)]$Cl_2$, the gold(III) analogue of [PtCl(Terpy)]Cl, originally characterized by Lippard /18/, was recently reported by us to possess relevant cytotoxic properties /19/. By analogy with the case of [PtCl(Terpy)]Cl, [AuCl(Terpy)]$Cl_2$ may be believed to act as an intercalating agent. However the structural features of the binding of this complex to DNA are still controversial and crystallographic information is lacking due to intrinsic difficulties in obtaining crystals suitable for X-ray diffraction.

In the present paper we have further addressed this issue by investigating, comparatively, the interactions of the structurally related [PtCl(Terpy)]Cl and [AuCl(Terpy)]$Cl_2$ complexes (see Figure 1) with double stranded DNA.

The metal complexes/DNA interactions were analysed in solution, at physiological pH, by a variety of physicochemical techniques including spectrophotometry, circular dichroism, ultrafiltration, DNA melting and gel electrophoresis. The joint application of these methods offers a rather complete description of the reactions of either complex with the DNA double helix. To our surprise, despite their strict structural similarity, these two complexes turned out to produce largely different DNA interaction patterns.

### EXPERIMENTAL

### Materials

[AuCl(Terpy)]$Cl_2$ was synthesized as reported /20/. The purity of the compound was checked through elemental analysis and $^1$H-NMR spectroscopy. *Ct*-DNA and [PtCl(Terpy)]Cl•$2H_2O$ (98% purity) were purchased from the Sigma Chemical Company. All other reagents used in this study were of analytical grade

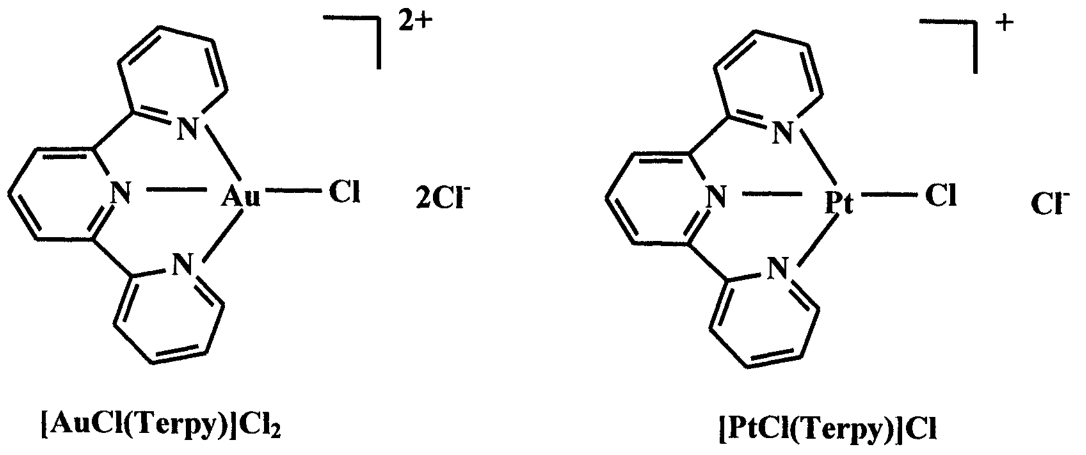

**Fig. 1:** Schematic drawings of $[AuCl(Terpy)]Cl_2$ and $[PtCl(Terpy)]Cl$.

and commercially available.

Plasmid pHV14, 7269 bp /21/, was prepared and purified using Qiagen Tip 500 (Qiagen Inc., Ca, USA); the preparation contained an average of 90 % supercoiled form.

### Spectrophotometric measurements.

Electronic absorption spectra were measured by means of a Perkin Elmer Lambda 20 Bio instrument. The reaction of the gold(III) complexes with *ct*-DNA was first analysed through absorption spectroscopy, since each complex has a characteristic spectrum in the UV-Visible region.

Electronic spectra of $[AuCl(Terpy)]Cl_2$ $5\times10^{-5}$ M and $[PtCl(Terpy)]Cl$ $2\times10^{-5}$ M were recorded before and after addition of *ct*-DNA (at r = 0.1, where r is the molar ratio between the added metal and DNA, expressed as base-pair) in the 50 mM phosphate, 4 mM NaCl, pH 7.4 buffer, at room temperature. The further evolution of the two systems over time was analysed until the spectral changes reached completion (data not shown).

In another set of experiments, visible absorption spectra were recorded at increasing complex/*ct*-DNA ratio (r = 0.0, 0.1, 0.2, 0.5, 1.0, 2.0 and 4.0) for $[AuCl(Terpy)]Cl_2$ and $[PtCl(Terpy)]Cl$ immediately after mixing, in the previous buffer, at room temperature.

### CD spectra

*ct*-DNA is characterized by two bands in the UV region: a positive peak at 260 nm and a negative one at 230 nm. We analysed the behaviour of the metal/DNA adducts at a fixed r during time, working in the 200-400 nm range. A *ct*-DNA solution in the reference buffer (50 mM phosphate and 4 mM NaCl, pH=7.4) was added to solutions containing $[AuCl(Terpy)]Cl_2$ $5\times10^{-5}$ M (to obtain r = 0.2) and $[PtCl(Terpy)]Cl$ $2\times10^{-5}$ M (r = 0.08).

To establish in more detail whether binding of the complexes brings about any significant conformational change of the DNA double helix, CD spectra of *ct*-DNA were recorded at increasing complex/*ct*-DNA ratios.

The CD technique is indeed very sensitive to detect minor conformational changes of the DNA conformation produced by ligand binding /22/.

CD spectra were recorded at increasing complex/*ct*-DNA ratio (r = 0.0, 0.1, 0.2, 0.5, and 1.0) for $[AuCl(Terpy)]Cl_2$ and [PtCl(Terpy)]Cl immediately after mixing. A Jasco J600 dichrograph operating at room temperature was used, interfaced with a PC, and spectra (in the 200-400 nm range) were analysed through the standard Jasco software package. *ct*-DNA concentration was $1.25x10^{-4}$M, 66 μg/ml in the reference buffer.

A second titration was carried out, with higher DNA concentration ($2.5x10^{-4}$M), to better analyse the "induced CD" bands, which appear in the presence of the metal complex.

### Ultrafiltration experiments

$1.25x10^{-4}$M DNA was dissolved in the physiological buffer and $[AuCl(Terpy)]Cl_2$ and [PtCl(Terpy)]Cl were added to obtain a r = 0.1 complex/*ct*-DNA ratio. The obtained samples were ultracentrifugated till half volume immediately after mixing at room temperature.

Ultrafiltration measurements were performed using tubes for concentration and purification of biological samples "Centricon Centrifugal Filters YM-10" by Amicon Bio-separation, Millipore Corporation, USA, with a Centurion centrifuge working at 3500 rpm for 30' – 45'.

The spectra of the upper solution containing *ct*-DNA and the bound metal, and of the lower solution, containing only the metal complex, were recorded using electronic absorption spectroscopy.

Afterwards, the amount of free and of DNA-bound metal was determined, by the electronic spectra, and the affinities of the individual complexes for DNA were qualitatively estimated.

### DNA melting studies

The effects of binding of the complexes on the stability of DNA double helix were investigated through analysis of the helix-to-coil transition. Melting experiments were carried out according to the reported procedure /23/. Renaturation profiles, recorded by progressive cooling of the sample, allow establishment of the formation of interstrand crosslinks, which favour the re-association process.

All melting measurements were carried out in the $10^{-2}$ M $NaClO_4$ and $10^{-3}$ M NaCl buffer. *Ct*-DNA was dissolved in the buffer and DNA concentration determined by absorption measurements at 260 nm. DNA concentration was equal to $4.9\times10^{-5}$ M [base pairs].

We recorded the melting curves (denaturation and renaturation) of the samples with r = 0.1 metal complex/DNA ratio.

Thermal denaturation experiments were performed in quartz cuvettes with a Perkin Elmer Lambda 20 Bio UV/Vis spectrophotometer equipped with a thermostated cell and a Peltier for temperature-control. Samples were continuously heated with a scan rate of 0.5°C/min while monitoring the absorbance changes at 260 nm. The investigated interval of temperature ranged from 45°C to 90°C. Upon reaching 90°C, samples were cooled back to 40°C in order to follow the renaturation process. Values for the melting temperatures ($T_m$) and the melting interval ($\Delta T$) were determined according to the reported procedures /24/.

### Gel electrophoresis.

Adducts with pHV14 plasmid DNA were prepared by adding the required volume of a freshly prepared solution of either [AuCl(Terpy)]$Cl_2$ or [PtCl(Terpy)]Cl in 50 mM sodium phosphate and 4 mM sodium chloride (pH 7.4) buffer. The concentration of pHV14 DNA in the reaction mixture was 75 ng/µl ($6x10^{-4}$M), while the concentration of the metal complexes varied to give different metal-to-bp stoichiometries (0.1, 0.2, 0.4 and 0.8). The mobility of the [AuCl(Terpy)]$Cl_2$ and [PtCl(Terpy)]Cl treated pHV14 samples was analyzed by gel electrophoresis /25/ on 0.8 % (w/v) agarose gel (Boehringer-Mannheim, Germany) at 10 V/cm at 4 °C for 8 h in Tris-acetate-EDTA buffer (TEA1X, pH=8), and then the gel was stained for 1 h in 0.5 µg/ml (w/v) of ethidium bromide. Later on, the gel was washed twice with the TEA1X buffer. The bands were photographed and analyzed with an UVP gel scanner (GDS2000; Ultra Violet Product Ltd., Cambridge, UK).

### RESULTS

### The absorption spectra

The reactions of [AuCl(Terpy)]$Cl_2$ and [PtCl(Terpy)]Cl with *ct*-DNA were first analysed by absorption spectroscopy in the visible spectra. Remarkably, both complexes are characterized by a number of intense transitions in the 300-400 nm region. It was previously shown that the visible spectra of both complexes, dissolved in a physiological buffer, are stable with time implying that the species existing in solution do not undergo further transformations /19/. Thus, samples of the two complexes were dissolved in the buffer and titrated with increasing amounts of *ct*-DNA. The spectral profiles of these titrations are shown in Figure 2. Addition of *ct*-DNA just induces a moderate hyperchromic effect on the visible spectrum of [AuCl(Terpy)]$Cl_2$, probably arising from the interference of *ct*-DNA with the intrinsic stacking processes of this gold(III) complex. In contrast, marked changes of the visible bands of [PtCl(Terpy)]Cl are observed mainly consisting of a large hypochromic effect and a red shift of the main transition, in line with previous observations on similar systems /10,26,27/.

### Circular dichroism

Addition of either [PtCl(Terpy)]Cl or [AuCl(Terpy)]$Cl_2$ to calf thymus DNA, at r=0.1 produces modest changes of its characteristic CD spectrum; however, if the concentration of these metal complexes is increased to r=1, large changes of the CD spectra are observed (see Figure 3). Pairwise, addition of increasing amounts of either complex to *ct*-DNA solutions results in the appearance of characteristic "induced CD" bands in the 300-400 nm region (see Figure 4).

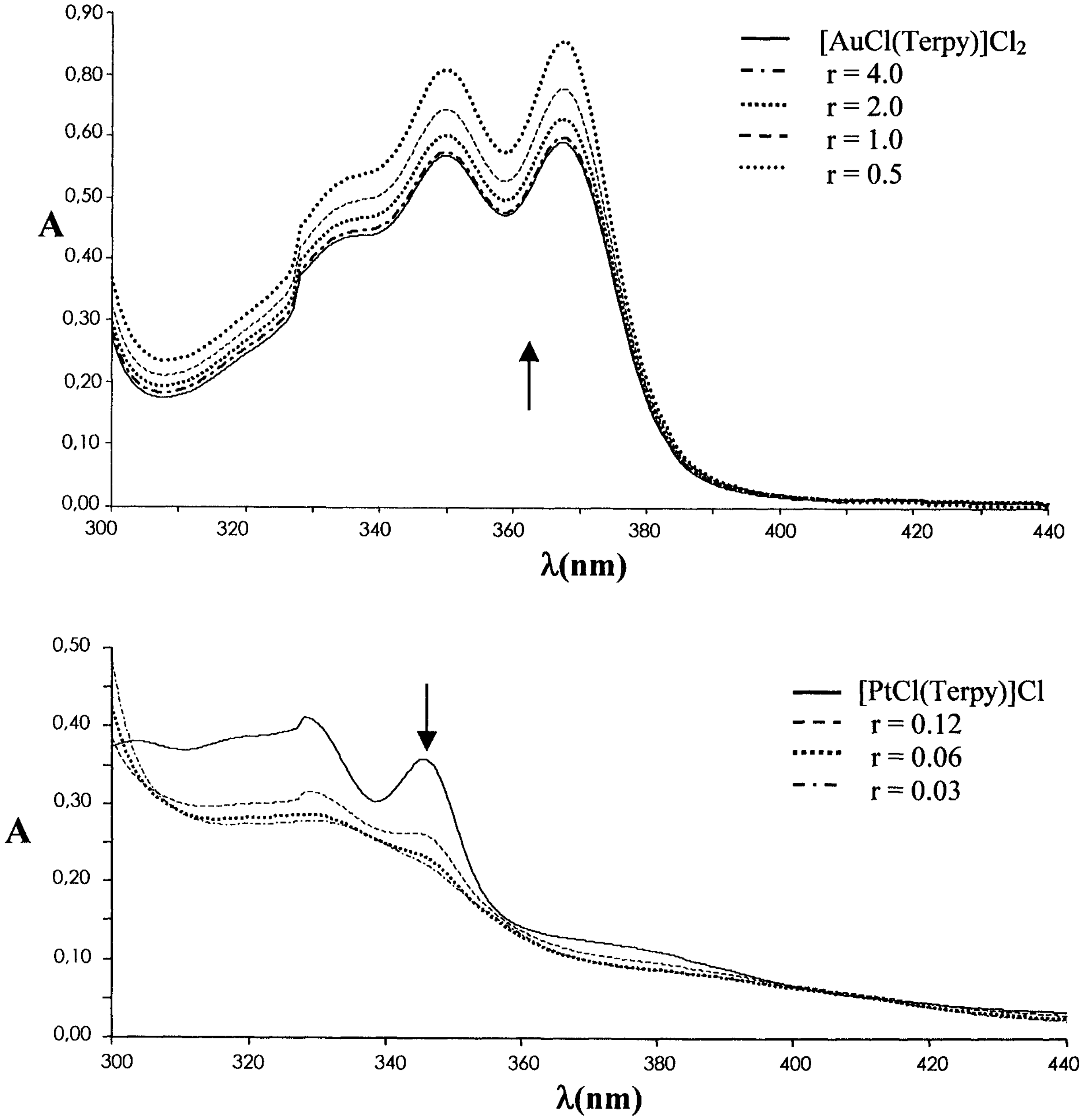

**Fig. 2:** The visible spectra of the two complexes before and after addition of *ct*-DNA at variable r in the reference buffer (50 mM $Na_2HPO_4$ and 4 mM NaCl, pH 7.4). $[AuCl(Terpy)]Cl_2$ concentration was 50 μM; [PtCl(Terpy)]Cl concentration was 20 μM.

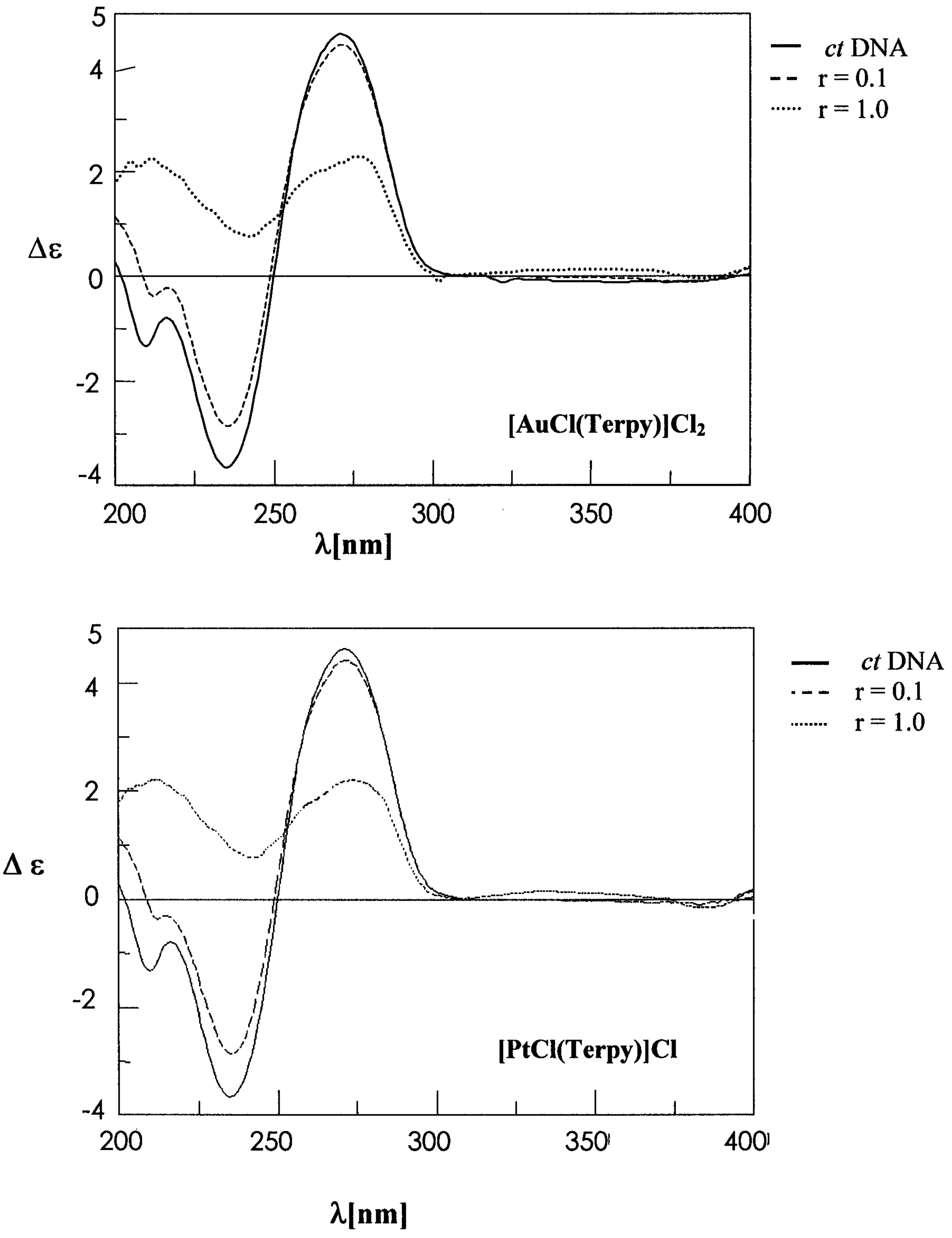

**Fig. 3:** CD titration of calf thymus DNA with increasing amounts of the two complexes; $[AuCl(Terpy)]Cl_2$ and [PtCl(Terpy)]Cl were added at the ratios: r=0.1, and 1.0 (from top to bottom on the positive band at 260 nm and the reverse on the negative band at 240 nm). DNA concentration was $1.25 \times 10^{-4}$M in base pairs.

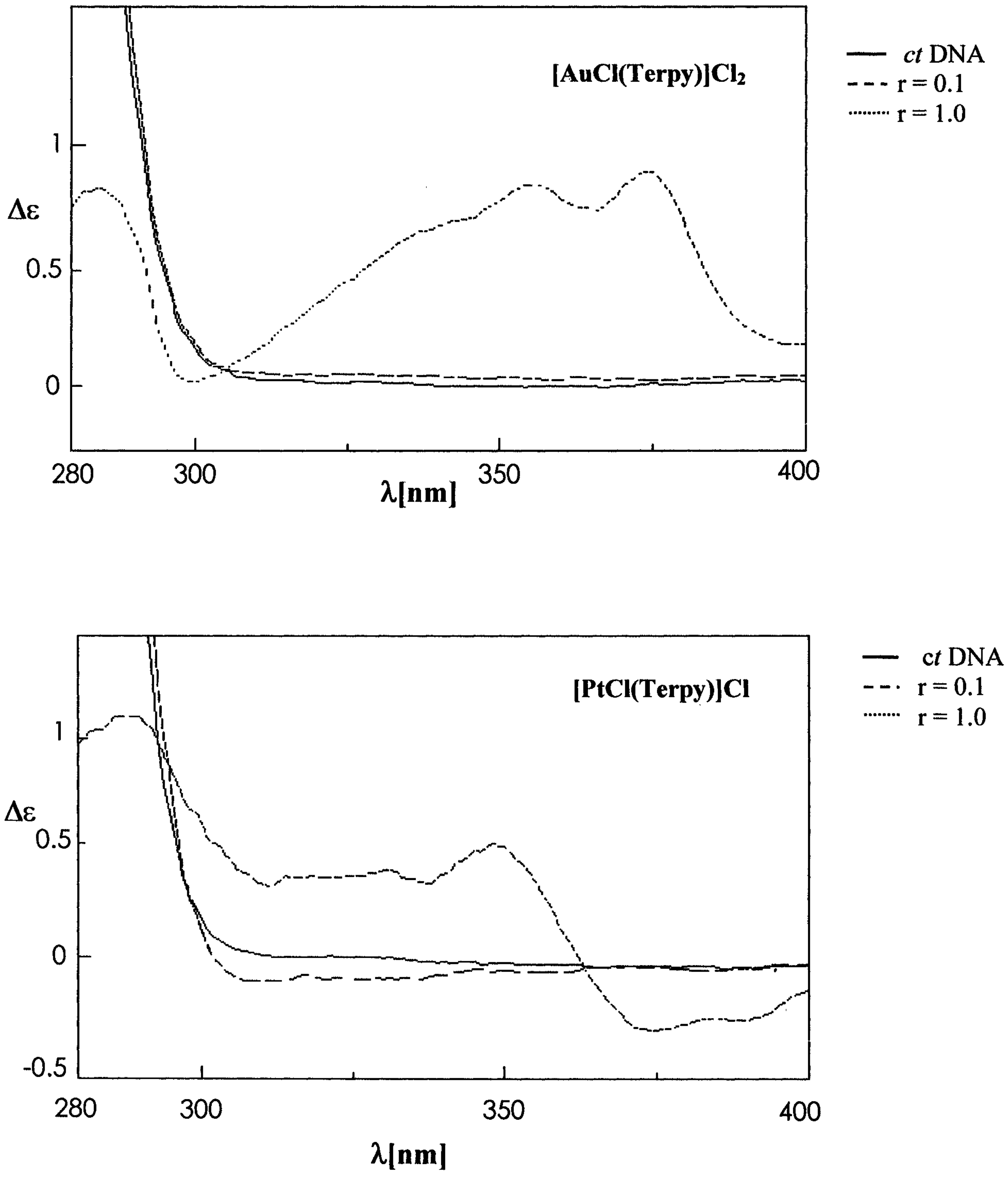

**Fig. 4:** CD titration of calf thymus DNA with increasing amounts of the two complexes; $[AuCl(Terpy)]Cl_2$ and [PtCl(Terpy)]Cl were added at the ratios: r=0.1, and 1. DNA concentration was $1.25 \times 10^{-4}$M (base pairs).

### Ultrafiltration studies

Solutions containing either [PtCl(Terpy)]Cl or [AuCl(Terpy)]$Cl_2$ in the presence of excess *ct*-DNA (r = 0.1), were ultrafiltered with a Centricon device and the content of the upper and lower solutions analysed by spectrophotometry. [PtCl(Terpy)]Cl is retained in the upper solution by *ct*-DNA far more strongly than [AuCl(Terpy)]$Cl_2$, indicating that the affinity of the former complex for *ct*-DNA is significantly greater with respect to [AuCl(Terpy)]$Cl_2$ (see Figure 5).

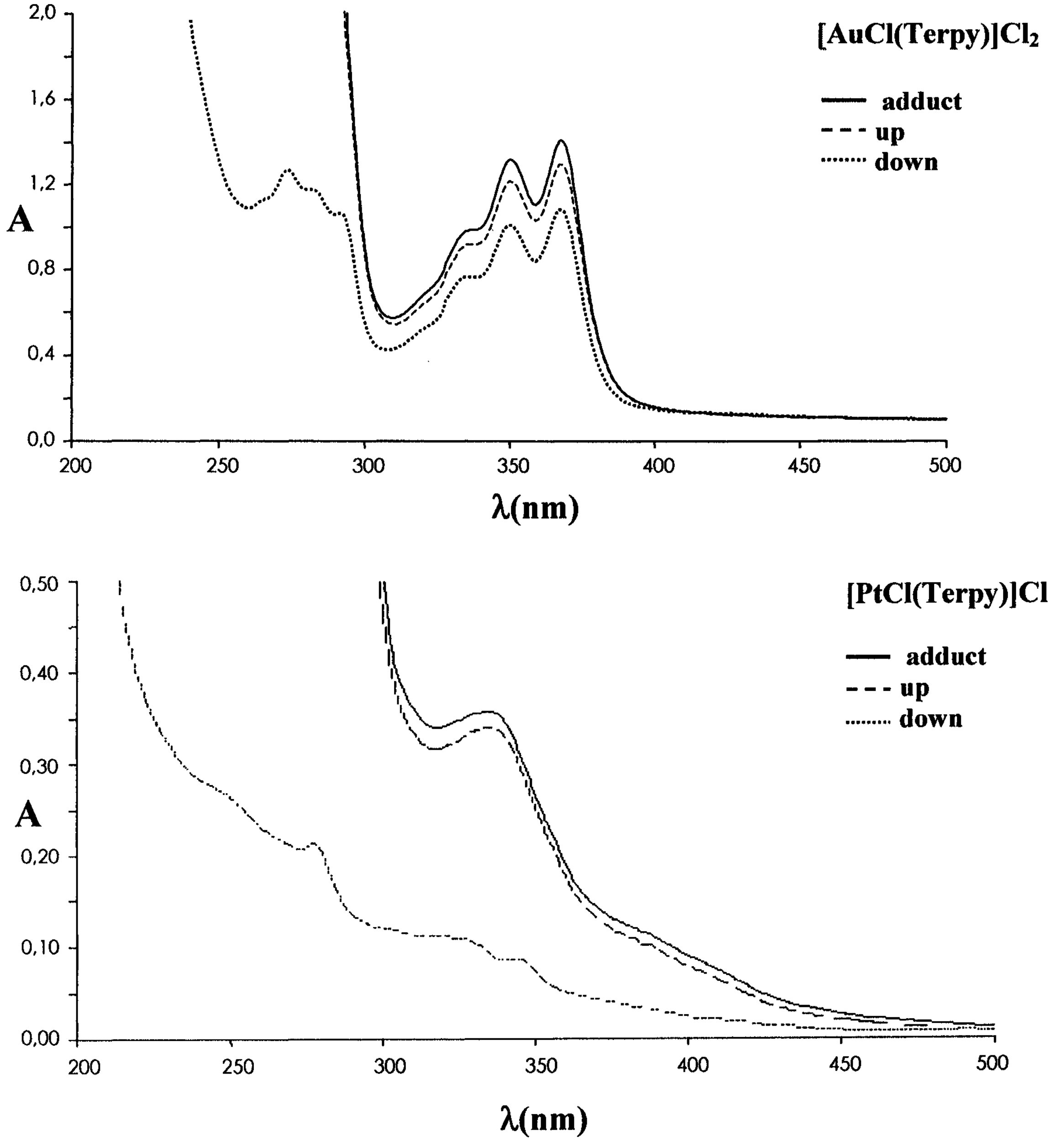

**Fig. 5:** The ultrafiltration experiments at half volume of the two metal compounds/*calf thymus* DNA adducts (r=0.1). Visible absorption spectra of the lower (down) and upper (up) solution were obtained after ultrafiltration (reducing the volume to half).

### Melting studies.

The consequences of adduct formation on the thermal stability of the double helix in *ct*-DNA were assayed by recording the DNA melting profiles. The effects of the two complexes on the DNA helix-to coil transition were analysed at fixed metal to bp ratio (0.1) immediately after mixing and after 24 hours of incubation (see Table 1 and Table 2). Addition of either compound produces a large thermal stabilization of the double helix. The measured increases in $T_m$ are +6.2°C (8.6°C after 24 hours) for [AuCl(Terpy)]$Cl_2$ and +3.2°C (2.7°C after 24 hours) for [PtCl(Terpy)]Cl.

**Table 1**

$T_m$, $\Delta T$, $\Delta H$, e $\Delta T_m$ values of metal complex/*ct*-DNA adducts (r=0.1), recorded at mixing at room temperature. $\Delta T_m$ is obtained by subtracting melting temperature of *ct*-DNA alone ($T_D$) from the melting temperature of the metal complex/*ct*-DNA adduct ($T_{Dc}$).

| | r | $T_m$ (°C) | $\Delta T$ (°C) | $\Delta H$ | $\Delta T_m$ (°C) |
|---|---|---|---|---|---|
| *ct*-DNA | 0.0 | 60.1 | 18.4 | 0.9 | – |
| DNA+ [AuCl(Terpy)]$Cl_2$ | 0.1 | 76.0 | 12.7 | 0.3 | 6.2 |
| DNA+ [PtCl(Terpy)Cl | 0.1 | 73.1 | 10.0 | 0.3 | 3.2 |

**Table 2**

$T_m$, $\Delta T$, $\Delta H$, e $\Delta T_m$ values of metal complex/*ct*-DNA adducts (r=0.1), recorded after 24 hours incubation at room temperature

| | r | $T_m$ (°C) | $\Delta T$ (°C) | $\Delta H$ | $\Delta T_m$ (°C) |
|---|---|---|---|---|---|
| *ct*-DNA | 0.0 | 69.9 | 18.4 | 0.9 | – |
| DNA+ [AuCl(Terpy)]$Cl_2$ | 0.1 | 78.5 | 12.5 | 0.3 | 8.6 |
| DNA+ [PtCl(Terpy)Cl | 0.1 | 72.7 | 11.8 | 0.3 | 2.7 |

### Gel electrophoresis experiments.

Representative gels are shown in Figure 6. Treatment of plasmidic DNA with [PtCl(Terpy)]Cl, using a short incubation time (1h), results in a marked decrease in the mobility of the supercoiled form, while [AuCl(Terpy)]$Cl_2$ is virtually ineffective on the electrophoretic profiles.

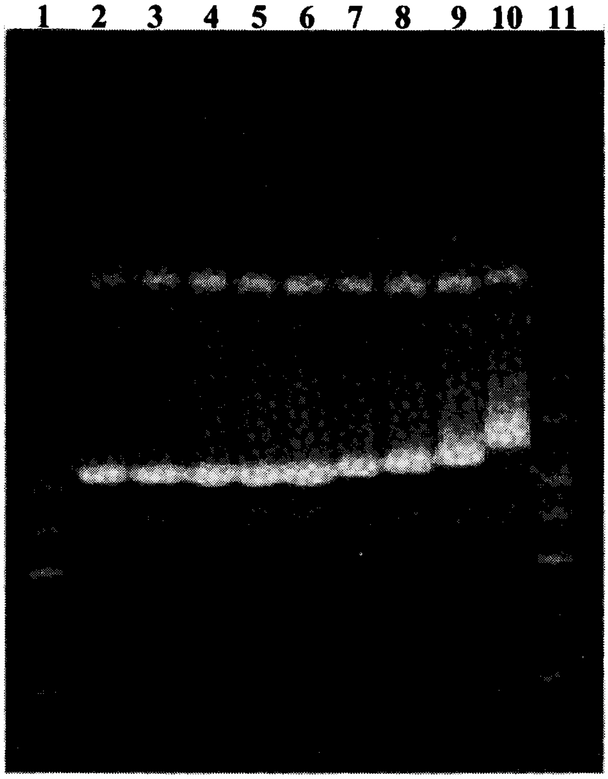

| | 1 | 2 | 3 | 4 | 5 | 6 | 7 | 8 | 9 | 10 | 11 |
|---|---|---|---|---|---|---|---|---|---|---|---|
| **[AuCl(Terpy)]Cl$_2$** | Ladder | pHV14 | 0.1 | 0.2 | 0.4 | 0.8 | | | | | Ladder |
| **[PtCl(Terpy)]Cl** | | | | | | | 0.1 | 0.2 | 0.4 | 0.8 | |

**Fig. 6:** Gel electrophoresis on 0.8% agar gel of the adducts of [AuCl(Terpy)]Cl$_2$ and [PtCl(Terpy)]Cl with plasmidic DNA (pHV14) in the buffer TEA1X (short incubation time t=1h).

The effects of the two complexes on the electrophoretic behaviour are strongly dependent on the incubation times. This prompted us to analyse gels of DNA-metal adducts at increasing time intervals after mixing. Results are shown in Figure 7. Electrophoretic retardation caused by [PtCl(Terpy)]Cl increased for increasing reaction times. The maximum effect, obtained after 10 hours of interaction, consisted of a remarkable retardation of plasmid mobility in the gel electrophoresis, similarly to what previously observed for cisplatin /28,29/. This behaviour is most likely attributed to formation of coordinative bonds. Notably, also in the case of [AuCl(Terpy)]Cl$_2$ some retardation in plasmidic mobility, though smaller than in the case of [PtCl(Terpy)]Cl, was observed for incubation times of 3 hours or larger (24 hours), again suggestive of formation of some coordinative bonds.

**1 2 3 4 5 6 7 8 9 10 11 12 13 14 15**

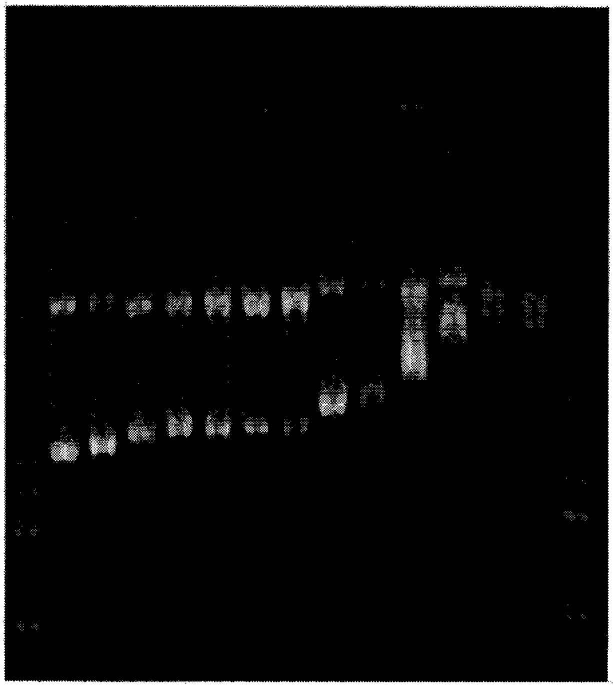

| | Incubation time | | | | | | | | | | | | | | |
|---|---|---|---|---|---|---|---|---|---|---|---|---|---|---|---|
| | **1** | **2** | **3** | **4** | **5** | **6** | **7** | **8** | **9** | **10** | **11** | **12** | **13** | **14** | **15** |
| **$[AuCl(Terpy)]Cl_2$** | Ladder | pHV14 | 0h | 1h | 3h | 5h | 10h | 24h | | | | | | | Ladder |
| **[PtCl(Terpy)]Cl** | Ladder | pHV14 | | | | | | | 0h | 1h | 3h | 5h | 10h | 24h | Ladder |

**Fig. 7:** Gel electrophoresis on 0.8% agar gel of the adducts of $[AuCl(Terpy)]Cl_2$ and [PtCl(Terpy)]Cl with plasmidic DNA (pHV14), r = 0.8, in the buffer TEA1X. A kinetic experiment.

### DISCUSSION

There is today great interest in characterising the interactions of metal complexes with nucleic acids in view of their relevant implications in medicine /1/. Indeed, much work has been done, and is still being done, to understand the structural reasons through which metal complexes are recognised by DNA molecules, to define the nature of binding and to comprehend the molecular bases of binding selectivity /1,17/. Unfortunately, crystallographic data on DNA/metal complexes adducts are very scarce due to the intrinsic difficulties in obtaining and growing crystals appropriate for diffraction studies. Thus, very often, only indirect structural information on these systems is available that is derived from the application of other physico-chemical techniques.

In this paper we have considered, comparatively, the interaction modes of two strictly related complexes [PtCl(Terpy)]Cl and [AuCl(Terpy)]$Cl_2$ to double stranded DNA. The two complexes are structurally similar in that both of them manifest a classical square planar geometry within a $d^8$ electronic configuration of the metal center /10,18/. The main formal difference is represented by the fact that the gold(III) complex possesses an additional positive charge. Moreover, other differences derive from the intrinsic differences in reactivity between platinum(II) and gold(III) complexes (*i.e.* gold(III) compounds are kinetically more labile, present pronounced oxidising properties and are far more acidic) /19/.

Notably, platinum terpyridine complexes have been the subject of several investigations during the last three decades, so that reliable models are available to describe their interactions with DNA /3-15/. At variance, only limited information is available on the gold(III) analogue that was first characterised by the group of Lippard /18/; then considered in our laboratory as a possible gold(III)-based cytotoxic and antitumor agent /19,30/.

Given the strict structural similarity of [AuCl(Terpy)]$Cl_2$ and [PtCl(Terpy)]Cl we proposed to establish whether such structural relatedness might result in a similar pattern of interactions with DNA.

An array of physicochemical methods has been utilised to gain information on the reaction of these metal terpyridine complexes with double stranded DNA, comprising visible spectrophotometry, circular dichroism, DNA melting analysis, ultrafiltration and gel electrophoresis.

Putting together the various pieces of information coming out from the different techniques the following picture emerges. Surprisingly, the two complexes show a pattern of interaction with DNA that is clearly distinct. In the case of [PtCl(Terpy)]Cl our data are in substantial agreement with previous reports from other laboratories /3-10/. The large variations of the visible absorption spectra observed at mixing, the profound effects produced by [PtCl(Terpy)]Cl on plasmidic DNA mobility and its tight binding to calf thymus DNA are fully consistent with the intercalation model proposed by Lippard /3-7/. Moreover, in line with previous observations /10/, we can postulate that the interaction of [PtCl(Terpy)]Cl with DNA progressively shifts with time from intercalation to formation of coordinative bonds. The time dependent changes of the gel electrophoresis patterns strongly support this interpretation.

Unexpectedly, [AuCl(Terpy)]$Cl_2$ exhibits a different interaction profile with double stranded DNA. Indeed, at mixing, there are modest changes in the visible spectra consisting of modest hyperchromic effects. Also, the binding affinity to calf thymus DNA is modest and the modifications of the gel electrophoresis patterns very small. These findings suggest that [AuCl(Terpy)]$Cl_2$ *is not able* to produce intercalative binding to DNA. This behaviour is rather surprising if one considers the tight structural similarity of the two complexes, the only major difference being the additional positive charge of the gold(III) analogue. Overall, the observed effects are consistent with external binding of the gold(III) complex to double stranded DNA mediated by electrostatic interactions between the positively charged complex and the negatively charged DNA double helix. Notably, with time, some differences appear in the interaction pattern of [AuCl(Terpy)]$Cl_2$ with DNA that, in analogy to the case of [PtCl(Terpy)]Cl, might be interpreted in terms of a progressive shift from external binding to formation of coordinative bonds. In line with this interpretation, the effects caused by [AuCl(Terpy)]$Cl_2$ on the gel electrophoresis profiles of plasmidic DNA become more marked.

**ACKNOWLEDGEMENTS**

The Cassa di Risparmio di Firenze and MIUR are gratefully acknowledged for a generous grant. We thank Dr. Daniela Vullo for helping us in the experimental work.

**REFERENCES**

1. S.J. Lippard and J.M. Berg, *Principles of Bioinorganic Chemistry*. University Science Books, 1994.
2. B. Norden, P. Lincoln, B. Åkerman and E. Tuite, *Met. Ions Biol. Syst.*, **33** (1997).
3. K. Becker, C. Herold-Mende, J.J. Park, G. Lowe and R.H. Schirmer, *J. Med. Chem.*, **44**, 2784-2792 (2001).
4. M. Howe-Grant, K.C. Wu, W.R. Bauer, S.J. Lippard., *Biochemistry*; **15**, 4339 (1976).
5. M. Howe-Grant, S.J. Lippard, *Biochemistry*, **18**, 5762 (1979).
6. J.K. Barton , S.J. Lippard , *Biochemistry*, **18**, 2661 (1979).
7. K.W. Jennette, S.J. Lippard, G.A. Vassiliades, W.R. Bauer, *Proc Natl Acad Sci U S A*, **10**, 3839 (1974).
8. D. R. McMillin and J. J. Moore, *Coord. Chem. Rev.*, **229**, 113 (2002).
9. D. K. Crites, C. T. Cunningham and D. R. McMillin, *Inorg. Chim. Acta*, **273**, 346 (1998).
10. C.S. Peyratout, T.K. Aldridge, D.K. Crites, D.R. Mc Millin, *Inorg Chem.*, **34**, 4484 (1995).
11. G. Arena, G. Calogero, S. Campagna, L. Monsu Scolaro, V. Ricevuto, R. Romeo., *Inorg Chem.*, **37**, 2763 (1998).
12. G.Arena, L. Monsù Scolaro, R.F. Pasternack, R. Romeo, *Inorg. Chem.*, **34**, 2994 (1995).
13. M. Casamento, G. E. Arena, C. Lo Passo, I. Pernice, A. Romeo and L. Monsù Scolaro, *Inorg. Chim. Acta*, **275-276**, 242 (1998).
14. K. Becker, C. Herold-Mende, J.J. Park, G. Lowe and R.H. Schirmer, *J. Med. Chem.*, **44**, 2784 (2001).
15. G. Lowe, A.S. Droz, T. Vilaivan, G.W. Weaver, J.J. Park, L. Tweedale and L.R. Kelland, *J. Med. Chem.*, **42**, 3167-3174 (1999).
16. A. McCoubrey, H.C. Latham, P.R. Cook, A. Rodger and G. Lowe, *FEBS Lett.*, **380**, 73 (1996).
17. M. Cusumano, M.L. Di Pietro and A. Giannetto, *Inorg. Chem.*, **39**, 50-55 (2000).
18. L.S. Hollis and S.J. Lippard, *J. Am. Chem. Soc.*, **105**, 4293-4299 (1983).
19. L. Messori, F. Abbate, G. Marcon, P. Orioli, M. Fontani, E. Mini, T. Mazzei, S. Carotti, T. O'Connell and P. Zanello, *J. Med. Chem.*, **43**, 3541 (2000).
20. S.D. Ehrlich, *Proc. Natl. Acad. Sci.*, **75**, 1433 (1978).
21. D.M. Gray, R.L. Ratliff and M.R. Vaughan, *Methods in Enzymol*,. **211**, 389 (1992).
22. A. Rodger and B. Norden, *Circular dichroism and linear dichroism* **cap. 1-2**, Oxford University Press, (1997).
23. J. Basu, N. Padhy and A. Mookerjee, *Indian J. Biochem. Biophys.*, **27**, 202 (1990).
24. W.D. Wilson, F.A. Tanios, M. Fernandez-Saiz and C.T. Rigl, *Methods Mol. Biol.*, Humana Press NJ, **90**, 219 (1997).

25. J. Sambrook, E.F. Fritsch and T. Maniatis, *Molecular Cloning - a Laboratory Manual*, Cold Spring Harbor Laboratory Press, (1989).
26. J.A. Todd and L.M. Rendina, *Inorg Chem.*, **41**, 3331 (2002).
27. H. Kurosaki, N Yamakawa, M. Sumimoto, K. Kimura, and M.Goto, *Bioorg. Med. Chem. Lett.*, **13**, 825 (2003).
28. G.L. Cohen, W.R. Bauer, J.K. Barton and S.J. Lippard, *Science*, **203**, 1014 (1979).
29. T.D. Tullius and S.J. Lippard, *Proc. Natl. Acad. Sci.*, **79**, 3489 (1982).
30. L. Messori, P. Orioli, C. Tempi and G. Marcon *Biochem. Biophys. Res. Commun.* **281**, 352 (2001).

